# Response to Letter to the Editor Regarding: Knowledge and Awareness of Tourette’s Syndrome Among Teachers in Eastern Region, Saudi Arabia

**DOI:** 10.5334/tohm.1211

**Published:** 2026-05-06

**Authors:** Zainab Alwusaybie, Mashael AlQuaimi

**Affiliations:** 1 College of Medicine, King Faisal University, Al-Ahsa, Saudi Arabia; 2 Department of Neurology, Movement Disorders Unit Prince Saud Bin Jalwi Hospital Al-Ahsa, Saudi Arabia

**Keywords:** Tourette syndrome, teacher knowledge, response to letter, Miller’s pyramid, Bloom’s cutoff

## Abstract

In this response to the Letter to the Editor regarding our article “Knowledge and Awareness of Tourette’s Syndrome among Teachers in Eastern Region, Saudi Arabia”, we address the correspondents’ methodological observations. We clarify that our questionnaire-based assessment was deliberately designed to measure declarative knowledge (the “knows” level of Miller’s pyramid) rather than applied classroom competence. We acknowledge the correspondents’ caution about dichotomising continuous variables using Bloom’s cutoff points and confirm that sensitivity analyses using continuous scoring yielded consistent conclusions. We thank the correspondents for their scholarly engagement and agree that future research should incorporate performance-based assessments and continuous scoring approaches.

Dear Dr. Louis,

We thank Dr. Sami Ullah Khan and Mr. Khan for their thoughtful commentary on our article, “Knowledge and Awareness of Tourette’s Syndrome among Teachers in Eastern Region, Saudi Arabia” [1]. We appreciate the opportunity to respond to the points raised and to further clarify aspects of our work.

We concur with the correspondents’ observation regarding the distinction between cognitive knowledge and applied classroom competence. As they correctly note, questionnaire-based assessments primarily capture the “knows” level of Miller’s pyramid [2]. Our study was deliberately designed to assess declarative knowledge and awareness as foundational elements, acknowledging that practical preparedness represents a higher-order construct requiring different methodological approaches. We were explicit in our manuscript that our findings reflect knowledge levels rather than teaching competence, and we appreciate the correspondents’ reinforcement of this important distinction for readers.

Regarding the application of Bloom’s cutoff points, we acknowledge the correspondents’ methodological caution about dichotomizing continuous variables. The referenced work by Altman and Royston [3] indeed raises valid concerns about information loss and potential misclassification at threshold boundaries. We selected Bloom’s method because it provides educators and policymakers with intuitively interpretable categories that facilitate intervention planning—for instance, identifying that 60.3% of participants fell below the 60% threshold conveys actionable information for educational authorities. Nevertheless, we agree that supplementary continuous analysis would enhance interpretation. Our dataset includes mean scores with standard deviations, and we are pleased to report that sensitivity analyses using continuous scoring (available upon request) yield consistent conclusions regarding knowledge gaps across demographic subgroups, mitigating concerns about threshold-driven artifacts.

We thank the correspondents for their scholarly engagement with our work. Their suggestions for future investigations incorporating performance-based assessments and continuous scoring approaches are valuable directions that would meaningfully extend this line of inquiry. We hope our response clarifies the deliberate methodological choices underlying our study while acknowledging the legitimate considerations raised.

Sincerely,

Zainab S. M. Alwusaybie, on behalf of all authors.
